# Educational outcomes of a medical physicist program over the past 10 years in Japan

**DOI:** 10.1093/jrr/rrx016

**Published:** 2017-04-11

**Authors:** Noriyuki Kadoya, Kumiko Karasawa, Iori Sumida, Hidetaka Arimura, Yasumasa Kakinohana, Shigeto Kabuki, Hajime Monzen, Teiji Nishio, Hiroki Shirato, Syogo Yamada

**Affiliations:** 1 The Japanese Board for Medical Physicist Qualification, Tokyo, Japan; 2 Department of Radiation Oncology, Tohoku University Graduate School of Medicine, 1-1 Seiryo-machi, Aoba-ku, Sendai 980-8574, Japan; 3 Department of Radiation Oncology, Tokyo Women's Medical University, Tokyo, 162-8666Japan; 4 Department of Radiation Oncology, Osaka University Graduate School of Medicine, Suita 565-0871, Japan; 5 Department of Health Sciences, Faculty of Medical Sciences, Kyusyu University, Fukuoka 812-8582, Japan; 6 Graduate School of Medicine, University of the Ryukyus, Okinawa 903-0213, Japan; 7 School of Medicine, Tokai University, Isehara 259-1193, Japan; 8 Department of Medical Physics, Graduate School of Medicine, Kindai University, Osaka-Sayama 589-8511, Japan; 9 Department of Medical Physics, Graduate School of Medicine, Tokyo Women's Medical University, Tokyo, 162-8666, Japan; 10 Department of Radiation Medicine, Hokkaido University Graduate School of Medicine, Sapporo 060-8638, Japan

**Keywords:** medical physicist, radiotherapy, education, career path, graduate school

## Abstract

The promotion plan for the Platform of Human Resource Development for Cancer (Ganpro) was initiated by the Ministry of Education, Culture, Sports, Science and Technology of Japan in 2007, establishing a curriculum for medical physicists. In this study, we surveyed the educational outcomes of the medical physicist program over the past 10 years since the initiation of Ganpro. The Japan Society of Medical Physics mailing list was used to announce this survey. The questionnaire was created by members of the Japanese Board for Medical Physicist Qualification, and was intended for the collection of information regarding the characteristics and career paths of medical physics students. Students who participated in the medical physics program from 2007 to 2016 were enrolled. Thirty-one universities (17 accredited and 14 non-accredited) were represented in the survey. In total, 491, 105 and 6 students were enrolled in the Master's, Doctorate and Residency programs, respectively. Most students held a Bachelor's degree in radiological technology (Master's program, 87%; Doctorate program, 72%). A large number of students with a Master's degree worked as radiological technologists (67%), whereas only 9% (*n* = 32) worked as medical physicists. In contrast, 53% (*n* = 28) of the students with a Doctorate degree worked as medical physicists. In total, 602 students (from 31 universities) completed the survey. Overall, although the number of the graduates who worked as medical physicists was small, this number increased annually. It thus seems that medical institutions in Japan are recognizing the necessity of licensed medical physicists in the radiotherapy community.

## INTRODUCTION

Cancers are among the leading causes of morbidity and mortality worldwide, with ~14.1 million new cases of cancer and ~8.2 million cancer-related deaths in 2012 [1]. In Japan in 2012, the number of new cases of cancer was ~865 000, and the number of cancer-related deaths was ~361 000 [2, 3]. To cultivate the human resources lacking in the clinical field of cancer, the Promotion Plan for the Platform of Human Resource Development for Cancer (Ganpro) was initiated by the Ministry of Education, Culture, Sports, Science and Technology of Japan in 2007 [4]. Eighteen groups, representing 94 universities nationwide in Japan, participated in this program to create an educational program designed to increase the number of graduate students majoring in medical oncology, radiation oncology, and palliative medicine, as well as cancer nursing, medical physics, cytology, and cancer pharmacy. For the medical physics program, 32 universities established a curriculum for medical physicists. In addition, the Japanese Board for Medical Physicist Qualification (JBMP) began accreditation of Master's, Doctoral and Residency programs for medical physics in 2012 [5]. Almost 10 years have passed since the initiation of Ganpro. To review the results of the educational project created by Ganpro, we need to clarify the educational outcomes of the medical physics program during this period. According to the JBMP, the number of certified medical physicists in Japan has increased annually from 140 in 2002 to 959 in 2016 [5]. The number of medical physicists per person (total number of medical physicists per national population) was 7.58 × 10^−6^ in Japan (959/126 541 000, 2016), whereas comparative numbers in the USA and Australia were 1.20 × 10^−5^ (3636/302 546 000, 2007) and 1.22 × 10^−5^ (274/22 520 000, 2011), respectively [5–7]. This result indicated that the number of medical physicists relative to the national population in Japan was still smaller compared with the numbers in other developed countries.

With regard to the clinical medical physicist in radiation oncology, the global medical physicist community has published a body of data. Chen *et al*. reported the results of the 2012 American Society for Radiation Oncology Workforce Survey, which mapped out the characteristics of the current radiation oncology workforce, including those of medical physicists [8]. Evans *et al*. reported the guidelines published by the Europe Federation of Organizations for Medical Physics regarding the roles, responsibilities and status of the medical physicist, together with recommended minimum staffing levels [9]. In the USA, medical physicists have commented on the need for a medical physicist assistant [10]. In addition, medical physicists have reported that future qualification as a qualified clinical medical physicist should be restricted to Doctoral degree holders [11]. Based on these papers, most countries would have trouble improving the education of medical physicists.

By understanding the educational outcomes of the current medical physicist program, we may then consider the next steps in seeking to further improve the education program for medical physicists in Japan.

In this study, we surveyed the educational outcomes of the medical physics program over the past 10 years.

## MATERIALS AND METHODS

The Japan Society of Medical Physics mailing list was used to announce this survey in September 2016 [12]. The questionnaire was created by a committee consisting of members of the JBMP. The questionnaire was intended for the collection of information regarding the characteristics and career paths of medical physics students. Students who participated in the medical physics program during the period from 2007 to 2016 were enrolled. The questions included: degree obtained (Bachelor, Master, or Doctorate), acquisition of national licensure for radiological technology, medical physicist certification, and career path of graduates.

## RESULTS

**Fig. 1.**
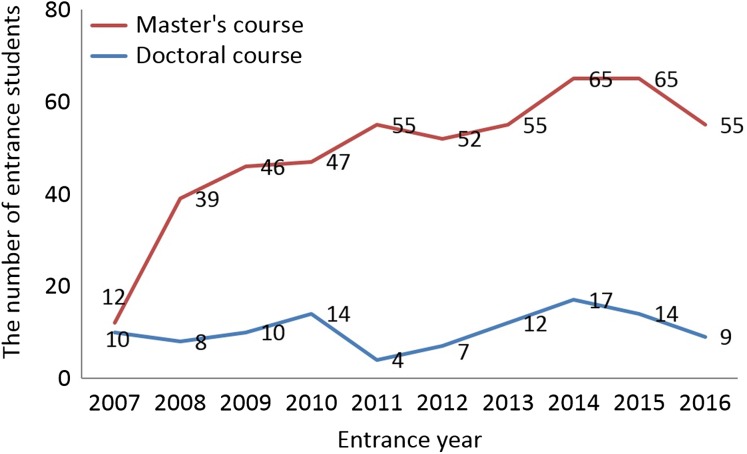
Number of entrance students to Master's and Doctoral programs for each entrance year.

A total of 31 responses were received: 17 responses from accredited universities and 14 responses from non-accredited universities. A total of 491, 105 and 6 students were enrolled in the Master's, Doctorate and Residency programs, respectively. Because of the small sample size, we were unable to perform a detailed analysis of the Residency program. Figure 1 shows the number of entrance students by entrance year. The numbers of students who participated in the Master's program were 12, 47 and 55 for 2007, 2010 and 2016, respectively, suggesting that the number of students who participated in the Master's program was increasing annually. For the Doctoral program, there was a constant number of students participating in the medical physics program each year (mean ± SD = 10.5 ± 3.6).

**Fig. 2.**
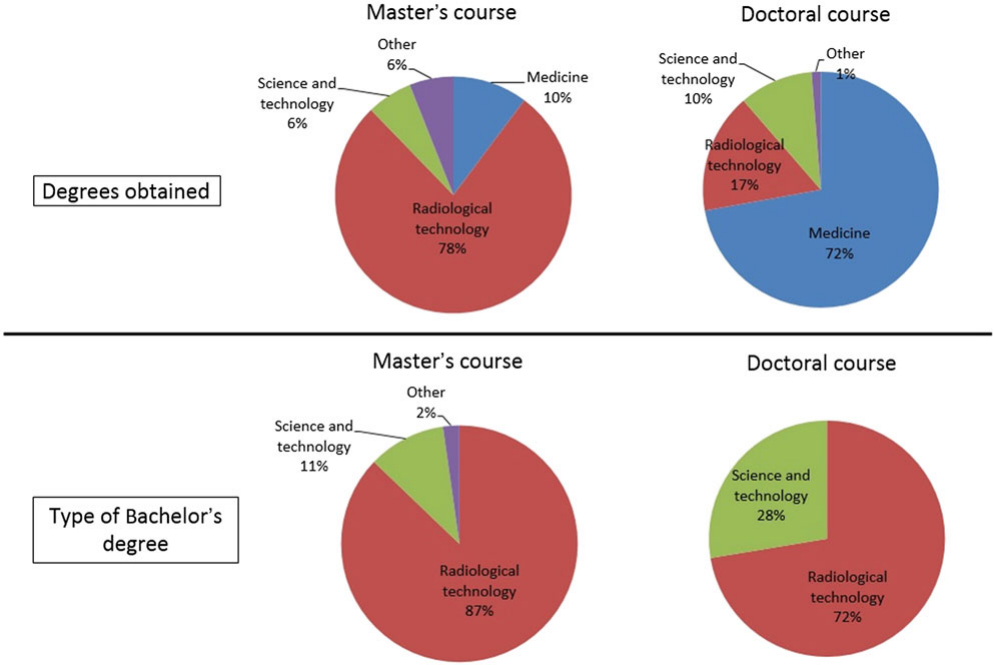
Pie charts showing degrees obtained (top figures) and type of Bachelor's degree (bottom figures).

**Fig. 3.**
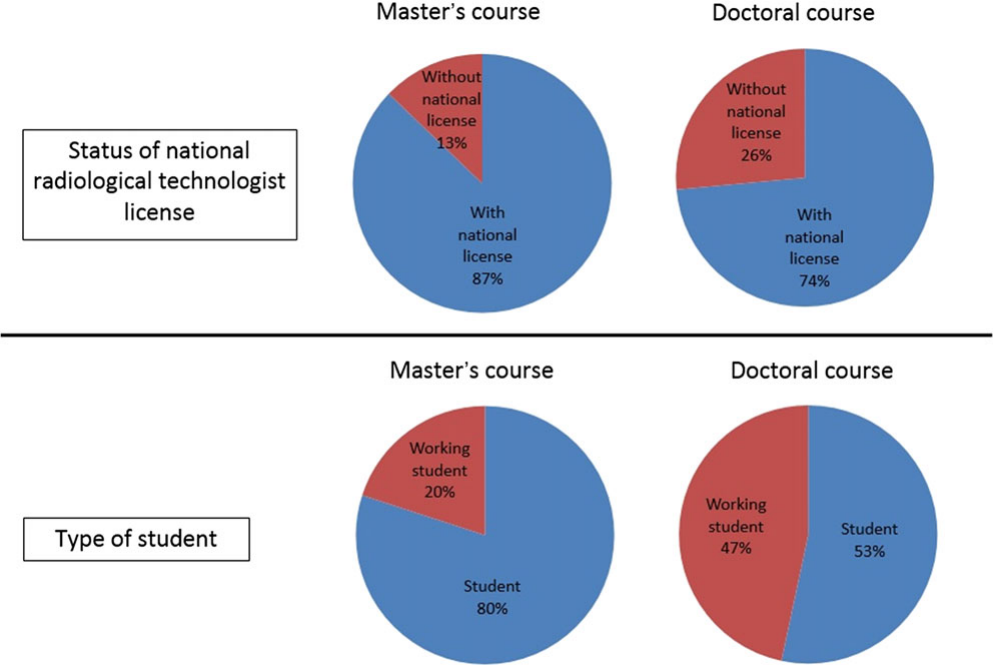
Pie charts showing status of national radiological technologist licensure (top figures) and type of student (bottom figures).

Figure 2 shows the data regarding the final degree obtained and type of Bachelor's degree. The majority (78%) of the students in the Master's program obtained a degree in radiological technology, whereas most (72%) in the Doctorate program obtained a degree in medicine. The majority of the students in both programs held a Bachelor's degree in radiological technology (Master's program, 87%; Doctorate program, 72%). Figure 3 shows the data regarding acquisition of a national radiological technologist license and type of student. The majority of students in both programs acquired a national radiological technologist license (Master's program, 87%; Doctorate program, 74%). Only 20% of the students in the Master's course were working students, but working students comprised 47% of students in the Doctoral course.

**Fig. 4.**
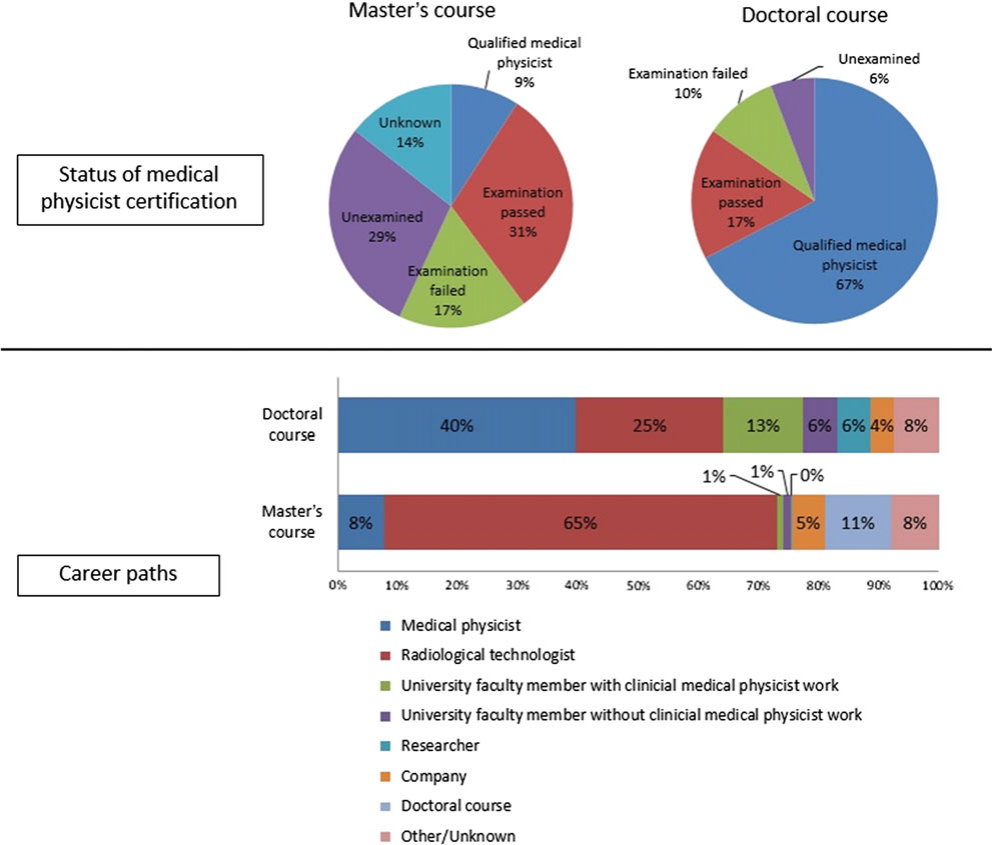
Pie charts showing the status of medical physicist certification (top figures) and bar charts showing career paths of graduates with Master's and Doctorate degrees (bottom figures).

**Fig. 5.**
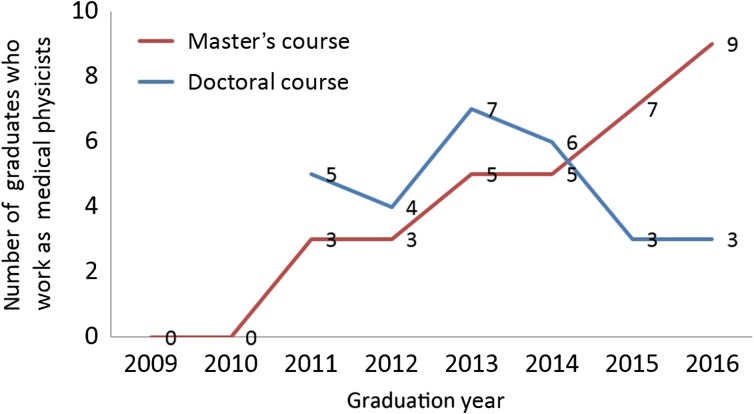
The number of graduates who worked as medical physicists for each graduation year.

**Fig. 6.**
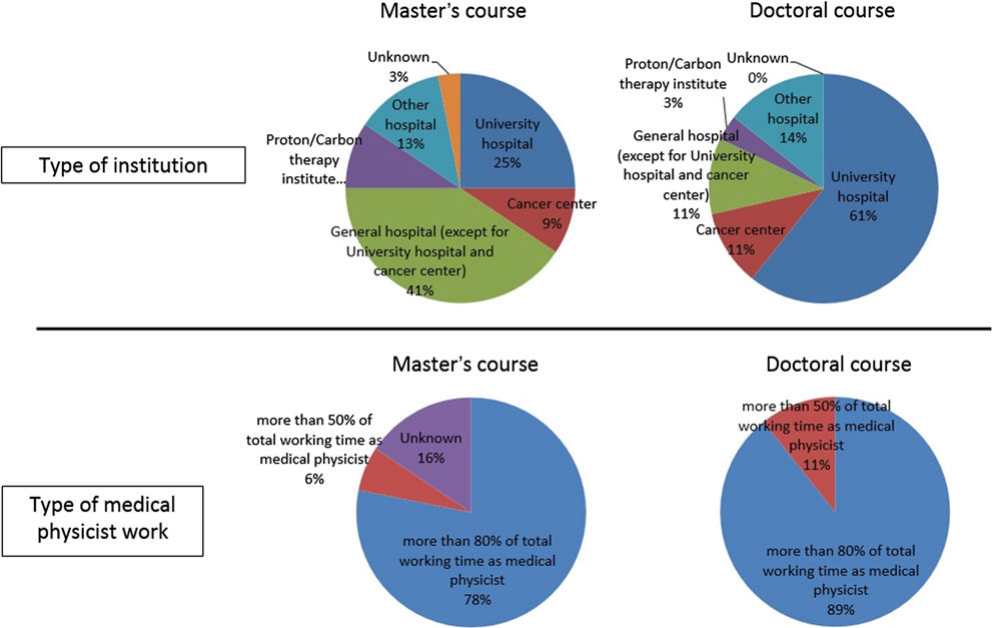
Bar charts showing the type of institution and type of medical physicist work.

The status of medical physicist certification and career paths are shown in Fig. 4. In terms of the acquisition of a medical physicist license, almost one-half of the graduates of the Master's program took the licensing examination (48%), and 65% of the graduates who took the examination passed. For graduates of the Doctorate program, 67% of graduates received a medical physicist license and 17% of graduates passed the examination. In terms of career paths of students following completion of the Master's and Doctorate programs, those with a Master's degree were most likely to work as radiological technologists (65%), whereas only 9% (*n* = 32) worked as medical physicists. In contrast, for Doctorate programs, the graduates who worked as radiological technologists were few (25%), and 53% (*n* = 28) of the graduates worked as medical physicists. In this analysis, the number of graduates who worked as medical physicists included the total number of medical physicists as well as university faculty members with clinical medical physicist work. Figure 5 shows the number of graduates who worked as medical physicists according to year of graduation. This result shows that the number of graduates with Master's degrees working as medical physicists significantly increased annually. In addition, a constant number of graduates with Doctoral degrees worked as medical physicists. The ratio of graduates working as medical physicists according to institution are shown in Fig. 6. Of those with a Master's degree, 41% worked in a general hospital (other than a university hospital or cancer center), whereas 61% of those with a Doctorate degree worked at a university hospital. Figure 6 also shows the proportion of students undertaking medical physicist work (>80% or >50%). This result clearly indicated that almost all medical physicists could focus on medical physicist work (Master's degree: 78%; Doctorate degree: 89%).

## DISCUSSION

In this study, for the first time we surveyed the educational outcomes of the medical physicist program over the past 10 years. Our results showed that a total of 491 and 105 students were enrolled in the Master's and Doctorate programs in the past 10 years, respectively. The number of students who participate in the medical physicist program is likely to continue to increase annually, indicating the continuing interest of undergraduate students. In addition, the number of students who worked as medical physicists had a tendency to increase annually (especially for those enrolled in the Master's program), showing that the medical institutions are gradually beginning to recognize the necessity of employing medical physicists in radiotherapy.

Based on the report of the number of enrollments in the medical physics program in the USA, Clark reported that there were ~1000 students enrolled in the medical physics program each year for the past 5 years (e.g. the number of newly enrolled students in 2015 was 1161), showing that the number of entrance students found in our study was significantly smaller than the number found by Clark [13]. In addition, medical physicists in the USA have reported that the American Association of Physicists in Medicine recognizes the need to reduce the number of graduate students (from ~250/year) to better align with the available Residency slots (~125/year) and manpower needs (~125/year). Requiring a Doctoral degree for a Residency aligns those graduates (~150/year) to the number of Residency slots [11]. Because of the large difference in the number of new patients treated with radiotherapy between Japan and the USA (i.e. 200 000 in 2009 in Japan vs 570 000 in 2004 in the USA), the required number of graduate students from the medical physics program would be smaller in Japan than in the USA. In the future, with an increasing number of slots for medical physicists and an increasing number of new patients in medical institutions, the number of graduate students should be increased (while giving attention to excessive supply—as in the USA) [14, 15]. A similar survey about the status of education and career paths of students after completion of the medical physicist program in Japan has already been conducted by the JBMP [16]. That survey was performed in August 2014. This previous study showed results similar to those of our study. They showed that almost all graduates (Master's degree, 87%; Doctorate degree, 74%) had a national radiological technologist license. In addition, they reported that 7% of those with a Master's degree and 50% of those with a Doctorate degree worked as medical physicists. This is consistent with our results. However, our study obtained a slightly higher percentage of graduates who worked as medical physicists, compared with the previous study (i.e. Master's course: 9% vs 7%; Doctoral course: 53% vs 50%). In addition, we found that the number of graduates who work as medical physicists is likely to continue to increase annually, especially for those enrolled in the Master's program. Hence, our study, with the latest data, was expected to indicate a higher percentage than the previous study from 2 years ago. Our result also supported the observation that medical physicists have gradually increased in number in the radiotherapy community in Japan.

There were more graduates employed as medical physicists who had a Master's degree than those who had a Doctoral degree. In Japan, to become licensed as a medical physicist, 2 years’ clinical experience is needed. Thus, although students enrolled in the Master's program can have taken the examination to become a medical physicist by the time they graduate, they cannot receive a license because they have no clinical experience. Since almost all job offers require a medical physicist license, graduates with a Master's degree might not be able to obtain a medical physicist job. In contrast, graduates with a Doctoral degree can receive the license by the time they have graduated and thus can obtain a medical physicist job. In addition, graduates with a Doctoral degree may have more motivation for becoming a medical physicist than those with a Master's degree. In the USA, only graduates of a Commission on Accreditation of Medical Physics Education Program (CAMPEP) accredited Residency program could enter the certification process of the American Board of Radiology (ABR) in 2014 [17]. After 2014, the minimum amount of post-baccalaureate training required to take the ABR examination will be ~2 years in a Master's course of the medical physics program, followed by 2 years of clinical training in a Residency program. In Japan, graduates with a Master's degree do not have clinical experience. Thus, it may be expected that graduates with a Master's degree who worked as medical physicists would be fewer than those with a Doctoral degree due to graduates with a Master's degree having no clinical experience. For further generation of professional medical physicists in the future, there must be an enhancement of the Residency program (there are just two Residency programs at present), similar to the program running in the USA. Kron *et al*. evaluated the status of medical physicists in the Asia–Pacific region [18, 19]. They found that 80% of medical physicists in Japan held a national radiological technologist license, which is consistent with our result. They also reported that most medical physicist tasks are performed by radiological technologists; they are well trained to do the job, but they are not called medical physicists. Our results indicated that the number of medical physicists working in medical institutions has gradually increased. In addition, almost all of the new medical physicists have worked >80% of their total working time as medical physicists. Based on these results, we can say that the radiotherapy community in Japan has gradually changed. That is, as in other countries, medical physicist tasks are likely to be performed by ‘medical physicists’ in Japan. Ganpro has effectively established the medical physicist in Japan. During the next decade, JBMP should seek to improve the quality of medical physicists and increase the number of medical physicists in the radiotherapy community.

## CONCLUSIONS

We surveyed the educational outcomes of the medical physicist program over the past 10 years for the first time. In total, 602 students from 31 universities completed the survey. Analysis of the survey results revealed that the majority of students (including those from Master's, Doctorate and Residency programs) held a Bachelor's degree in radiological technology. We also determined that the majority of Master's students worked as radiological technologists (vs medical physicists), whereas more than one-half of Doctoral students worked as medical physicists. Overall, although the number of the graduates who worked as medical physicists was small, this number increased annually. It thus seems that medical institutions in Japan are recognizing the necessity of having licensed medical physicists in the radiotherapy community.
